# Visualization and mapping of literature on the scientific analysis of wall paintings: a bibliometric analysis from 2011 to 2021

**DOI:** 10.1186/s40494-022-00735-0

**Published:** 2022-07-07

**Authors:** Zhanyun Zhu, Xiuya Yao, Yaling Qin, Zhiyong Lu, Qinglin Ma, Xi Zhao, Liu Liu

**Affiliations:** 1 Research and Practice Base of Conservation Science and Engineering, Department of History, College of Humanities, Xiamen University, Xiamen, 361005 China; 2 Department of Archaeology, Max Planck Institute for the Science of Human History, D-07745 Jena, Germany; 3 Key Scientific Research Base of Conservation and Restoration for Murals as Collection and Materials Science in State Administration for Cultural Heritage, Shaanxi History Museum, 710061 Xi׳‬an, China; 4 Joint International Research Laboratory of Environmental and Social Archaeology, Institute of Cultural Heritage, Shandong University, Qingdao, 266237 China; 5 Xiamen Academy of Arts and Design, Fuzhou University, Xiamen, 361000 China

**Keywords:** Wall painting, Bibliometric analysis, VOSviewer, Visual analysis

## Abstract

As non-renewable cultural heritage, wall paintings play an important role in society. To reveal the trends in the scientific analysis of mural paintings, 845 relevant research articles published from 2011 to 2021 were collected from the Web of Science database and analyzed. The VOSviewer software was adopted to map the network data of scientific publications, so that relationships among authors, countries, institutions can be displayed, and the co-occurrence of keywords and co-citation can be analyzed. The results revealed close and strong interconnections between the top authors, suggesting a considerable strong research link in this field. The cooperation between research institutions was relatively close. The most productive country of relevant publications was Italy. The leading journals for the scientific analysis of wall paintings were *Journal of Raman Spectroscopy* and *Journal of Cultural Heritage*. At present, the hotspots of scientific analysis and research on wall painting are revealing the composition, distribution, origin, and deterioration mechanism of pigments, alongside with evaluating the effects and mechanism of conservation materials and techniques. On the one hand, a possible development direction in this field is introducing more cutting-edge analysis and data processing methods. On the other hand, scientific analysis is increasingly adopted to guide the research and development of mural conservation materials.

## Introduction

Wall paintings are one of the earliest painting forms in human history, tracing back to the late Paleolithic period. The earliest examples include the mural of Chauvet Cave in Ardèche, southern France. Many other ancient murals were also preserved, including the ones found in the ancient tombs of the Valley of the Kings, the palaces of Crete civilization, and Pompeii [1]. The development of wall paintings is closely connected with the customs, religion, philosophy, and aesthetics of different nationalities in various historical periods. As precious non-renewable cultural heritage, the production of murals is also compatible with the political, economic, cultural, and technological development of certain societies at certain times [2].

Affected by natural and human factors, wall paintings were damaged to varying degrees over time. Following the principle of minimum intervention, analysis of wall paintings adopts various scientific and technological methods to investigate the materials and techniques of ancient murals, assess their preservation status, and prepare for future restoration and preventive conservation. The analysis of ancient mural samples began in 1800 when Haslam examined samples of English medieval wall paintings and characterized 6 different pigments [3]. In 1814, Davy analyzed the various pigments in murals from Rome [4]. With the development of chromatography and spectroscopy in the 1950s, traditional chemical methods have been gradually replaced by modern analytical methods [5]. In the mid-1980s, Guineau applied Raman spectroscopy to analyze murals [6, 7], which later played an important role in the mural analysis [8]. In the 1990s, FT-IR was recognized as a very useful instrumental technique in the analysis and technical examination of wall paintings [9]. It provides a method of analysis which does not require much complex preparation of samples. With technological advancements, many researchers in chemistry, biology, and materials science carried out scientific analysis of mural paintings, which promoted the continuous development of theories and technologies in this field and the accumulation of research articles [10–14].

There are more than 10 commonly used methods for the scientific analysis of wall paintings. Chemical analysis methods, X-ray fluorescence (XRF), Raman spectroscopy (RS), and polarized light microscopy (PLM) have been adopted to detect mural pigments and clarify their material composition. Pyrolysis gas chromatography mass spectrometry (Py-GC/MS), liquid chromatography mass spectrometry (LC/MS), proteomics, immunology, and other analytical techniques have been applied to analyze the binding materials. X-ray diffraction (XRD) and scanning electron microscopy (SEM) have been used to analyze the structure of the ground layer [15–20]. Comprehensive application of the various analytical methods can help determine the mechanisms of deteriorations and offer important theoretical support to develop targeted conservation measures.

Scientific analyses on wall paintings have been increasing in recent years. Bibliometric research can objectively and comprehensively reveal the development and trends in a field and help fellow researchers quickly understand the research focus. VOSviewer is developed by Nees Jan van Eck and Ludo Waltman of Leiden University in the Netherlands for mapping and visualizing econometric networks. It can display the development, research focus, and trends of a certain discipline within a certain period and reveal the evolution of multiple research frontiers [21].

In this paper, the relevant literatures published between 2011 and 2021 were collected from the Web of Science database, then bibliometrics and knowledge graph analysis were conducted on the VOSviewer software. The situation of scientific analysis on wall paintings in the past decade was visualized. The relevant literatures were quantitatively analyzed to form the corresponding knowledge map, identify the knowledge base of the research area, and provide the latest progress of related research, frontiers, hotspots, evolution paths, and future development trends of the scientific analysis of wall paintings. This study could promote further development in the scientific analysis of wall paintings.

## Methodology

The bibliometric analysis combines mathematics, statistics, and other measurement methods to study the distribution structure, quantitative relationship, and variation pattern of literature. In addition to articles and books, its analysis objects also include the relevant information within the article, such as the title, subject terms, keywords, word frequency, co-citation, co-occurrence, citation information, co-cited references, citation coupling, author, collaborator, publisher, date, language, institution, and country, thus can be used to analyze the research overview and development trend of a subject [22].

**Fig. 1:**
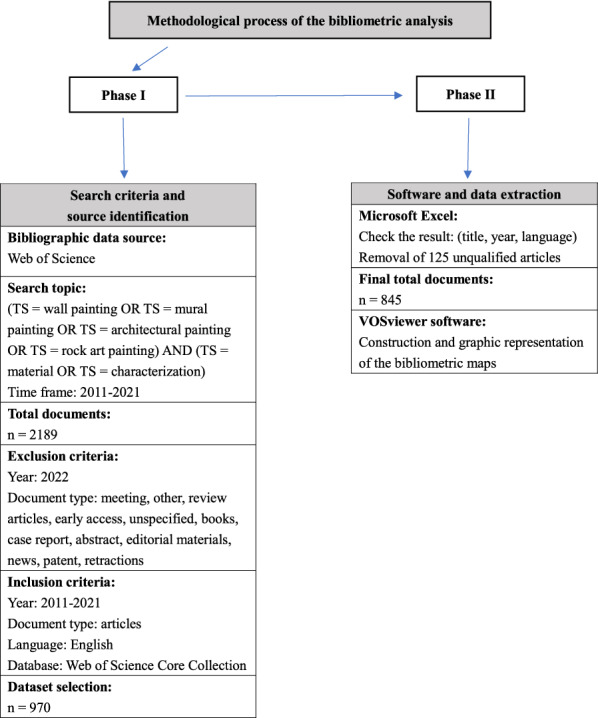
Flow chart of the search strategy

Taking the Web of Science database as the data source, the search formula of (TS = material OR TS = characterization) AND (TS = mural painting OR TS = wall painting OR TS = architectural painting OR TS = rock art painting) was adopted to collect articles published from 2011 to 2021. The article type filter was set to journal articles, and the language filter was set to English. The collected articles with Full Record and Cite References were then saved as plain text for subsequent analysis. The original data were imported into an Excel spreadsheet, and the year of publication, language, title, and article type of all the articles were checked manually. Items were rejected if they were not published in English, or without the 2011–2021 period, or on an irrelevant topic. The flow chart of the search strategy was shown in Fig. 1, and finally a dataset with 845 articles was formed.

The final dataset was imported into VOSviewer to obtain the bibliometric analysis graphs, where a circle and label represent a node, and a larger circle represents a higher level of importance. The same color signifies the same cluster. The node types in this study included authors, countries, institutions, journals, and keywords. The corresponding cooperation network analysis, co-citation analysis, and co-occurrence analysis were conducted.

## Results and discussion

### Analysis of publications

Changes in the number of published articles of a specific research direction directly reflect the variation of research outputs within a specific period. Therefore, it is an essential indicator of the development trend in that period. It is of great significance for analyzing the dynamics and trends of future research and development [23].

**Fig. 2:**
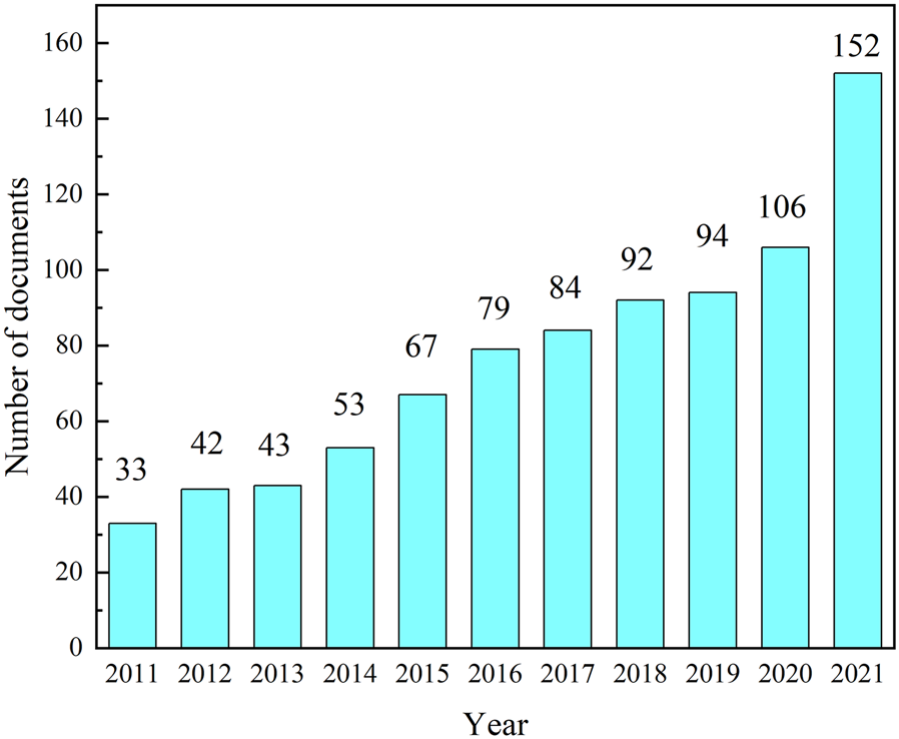
The number of published papers on scientific analysis of murals (2011–2021)

Figure 2 shows the evolution in the number of published articles and the significant variations within this research field during the whole study period. Overall, the number of publications in this field is increasing steadily year by year. The highest productivity was observed in 2021 with a total of 152 papers, whereas the lowest was in 2011 with a total of 33. It can be seen that the number of literatures increased rapidly from 2020 to 2021. The COVID-19 pandemic in 2020 may has a bearing on this situation, as it has led to the postponement of some cultural heritage projects till 2021.

### Article network analysis

#### Analysis of author cooperation relationship

**Fig. 3:**
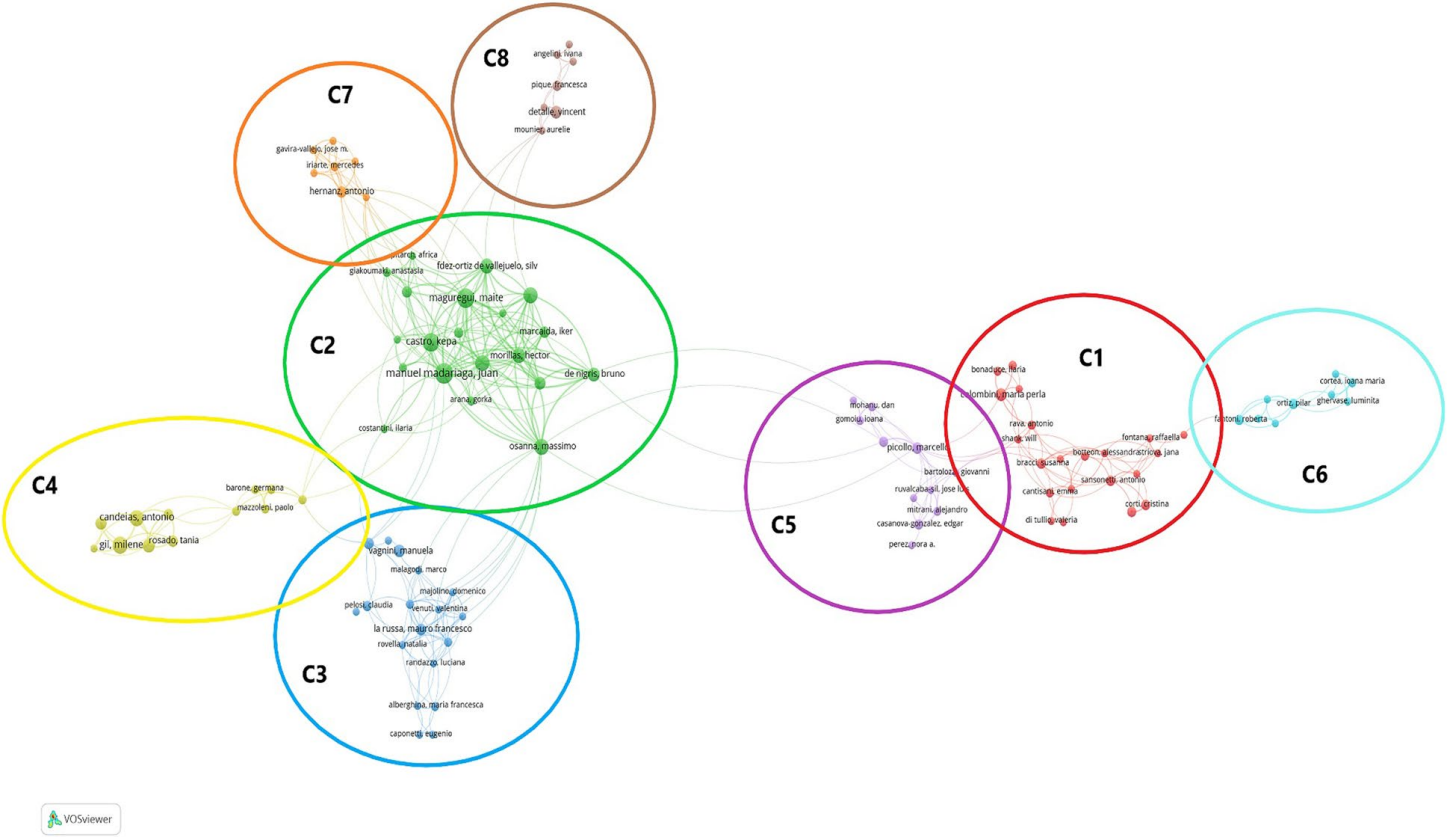
Co-authorship network of scientific analysis of murals (2011–2021)

The co-authorship network of publications in mural scientific analysis from 2011 to 2021 revealed 3279 authors. Under the threshold of at least 3 published articles per author with 3 citations per author, 199 authors were identified, but only 105 authors were visually mapped in Fig. 3 as some of them were not interconnected.

Citation is the most frequent method used as a measure of the influence of an author or a paper because it can quickly identify important works in the field [24, 25]. Table 1 showed the top 10 authors by citations, namely Maguregui, Madariaga, Castro, Martinez-Arkarazo, de Vallejuelo, Veneranda, Bersani, Detalle, Giakoumaki, and Osanna. Seven of the top 10 authors are from Spain, indicating that Spain has a major contribution to the scientific analysis of wall paintings. These influential authors mainly focused on chemistry, spectroscopy, and materials science.

As shown in Fig. 3, the lines among the authors represent their cooperation links, and the 8 different colors represent the author collaboration clusters. It can be noted that authors from the same country were closely related. There were also cross-border cooperations, such as Holakooei from Iran has a partnership with Casoli from Italy in the C3 cluster, Vandenabeele from Belgium has a partnership with Mirao from Portugal in the C4 cluster.

**Table 1:** Top 10 authors of mural scientific analysis (ranking by citations)

Ranking	Author	Country	Citations	Documents	Links	Total link strength
1	Maguregui, M	Spain	285	15	20	94
2	Madariaga, JM	Spain	285	17	22	97
3	Castro, K	Spain	263	14	21	69
4	Martinez-Arkarazo, I	Spain	169	6	11	24
5	de Vallejuelo, SFO	Spain	155	9	18	66
6	Veneranda, M	Spain	144	10	16	68
7	Bersani, D	Italy	127	4	8	10
8	Detalle, V	France	122	7	3	5
9	Giakoumaki, A	Spain	122	4	10	20
10	Osanna, M	Italy	112	10	22	71

Link strength is the number of publications two researchers have co-authored

#### Analysis of country cooperation

From 2011 to 2021, there were 76 countries involved in the research on the scientific analysis of murals. Under the threshold of at least 5 articles published per country with a minimum of 5 citations, 36 countries were selected, resulting in a country cooperation relationship map with 145 links and a total link strength of 374. The top 5 countries of relevant publications are Italy (236), Spain (142), China (82), USA (73), France (63).

**Fig. 4:**
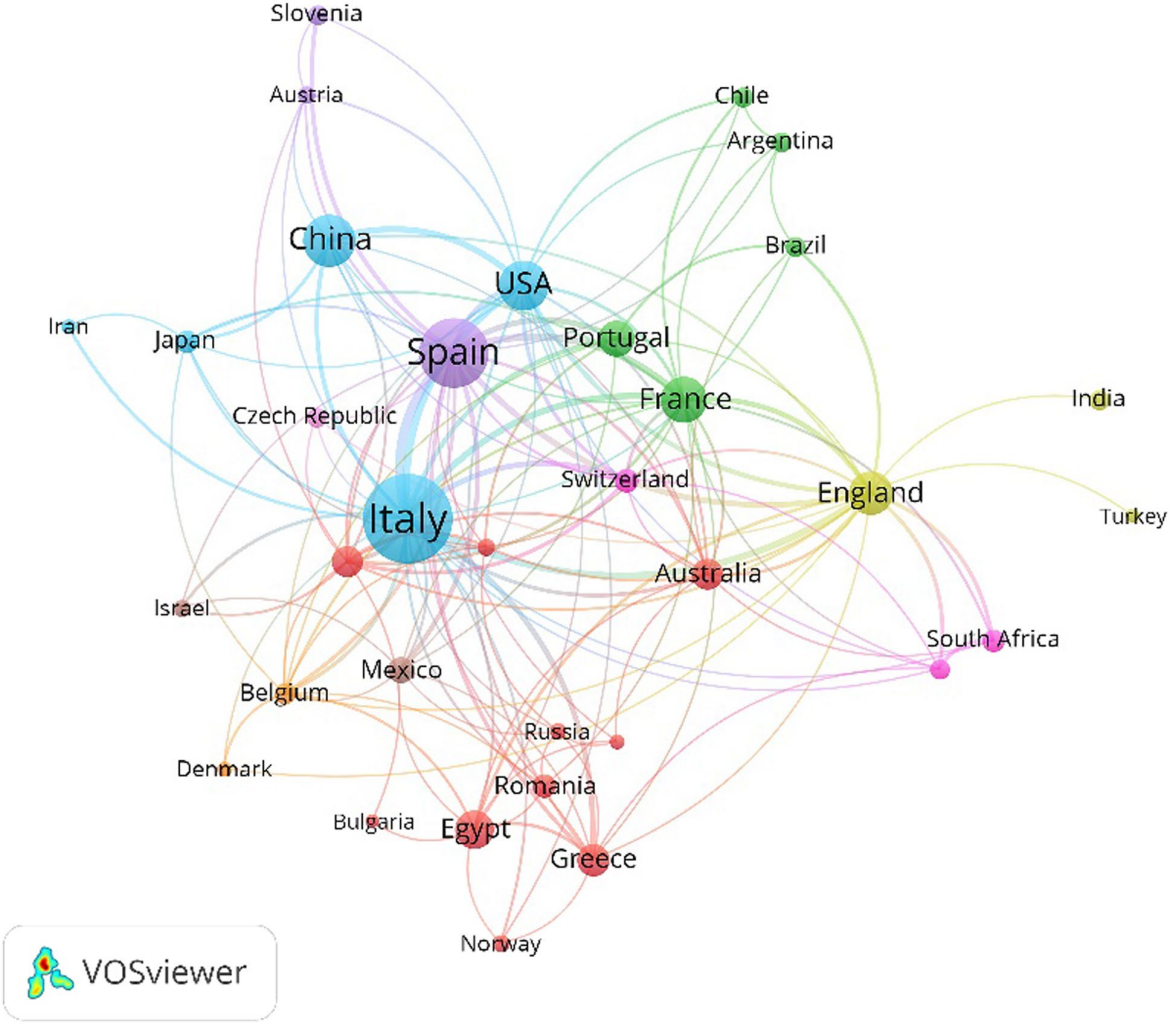
Co-authorship network map of countries publishing on scientific analysis of murals (2011–2021)

As shown in Fig. 4, each country is represented by a node sized proportionally to its number of publications. The lines connecting the nodes show the existing interconnection among the countries. The largest node diameters manifested by Italy and Spain indicate that they are the main countries in mural scientific analysis by absolute influence. This may be linked to the fact that both countries have the largest cultural heritage remains in Europe. Among the top 10 countries by node diameter, there are 8 countries from Europe, indicating that European scholars are leading in this field. As shown in Table 2, the country with the highest number of citations is Italy (3092), followed by Spain (1736) and France (978). Although China ranks third by the number of publications, its ranking is not high in the co-authorship network. The reason may be that Chinese scholars focused largely on domestic academic exchanges.

**Table 2:** Top 10 countries contributed to the scientific analysis of murals (ranking by articles)

Ranking	Country	Continent	Articles	Citations	Links	Total link strength
1	Italy	Europe	236	3092	26	126
2	Spain	Europe	142	1736	22	101
3	China	Asia	82	586	10	27
4	USA	America	73	560	19	61
5	France	Europe	63	978	18	58
6	England	Europe	57	629	20	66
7	Egypt	Africa	45	230	11	17
8	Portugal	Europe	38	351	7	22
9	Greece	Europe	31	435	11	24
10	Germany	Europe	29	237	14	31

Link strength is the number of publications two countries have co-authored

#### Institutional analysis

**Fig. 5:**
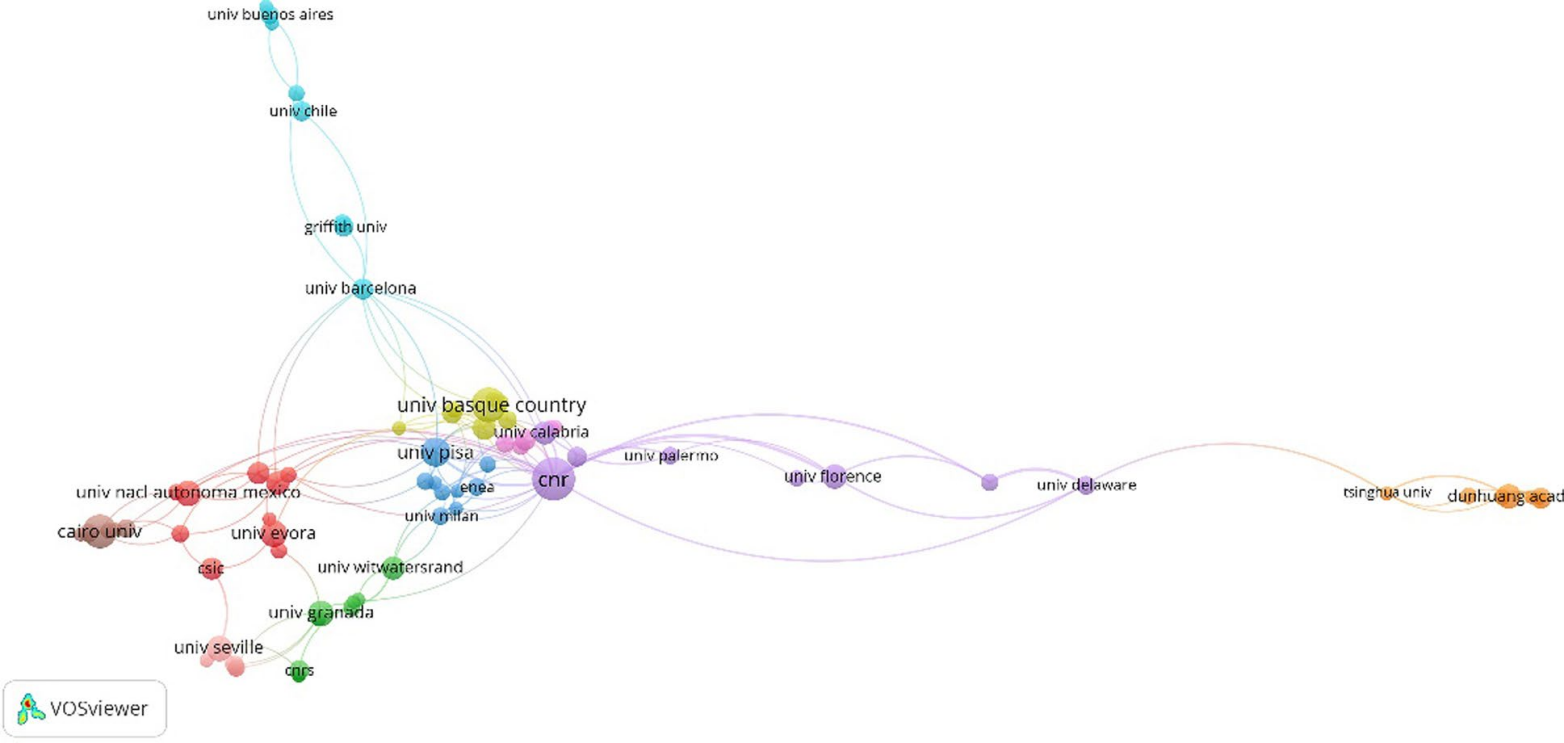
Co-institution network on scientific analysis of murals (2011–2021)

From 2011 to 2021, 1173 research institutions were involved in the scientific analysis of murals. A total of 79 research institutions reached the threshold of 5. However, only 76 institutions are shown on the map because some have no cooperative relationship. The number of links in the institution cooperation map is 165, and the total link strength is 272 (Fig. 5). Table 3 lists the top 10 research institutions in the scientific analysis of wall paintings: CNR (42), University of the Basque Country (28), Cairo University (26), University of Pisa (20), University of Evora (17), University of Granada (16), Universidad Nacional Autonoma de Mexico (15), University of Seville (15), Dunhuang Academy (14), and University of Florence (14).

**Fig. 6:**
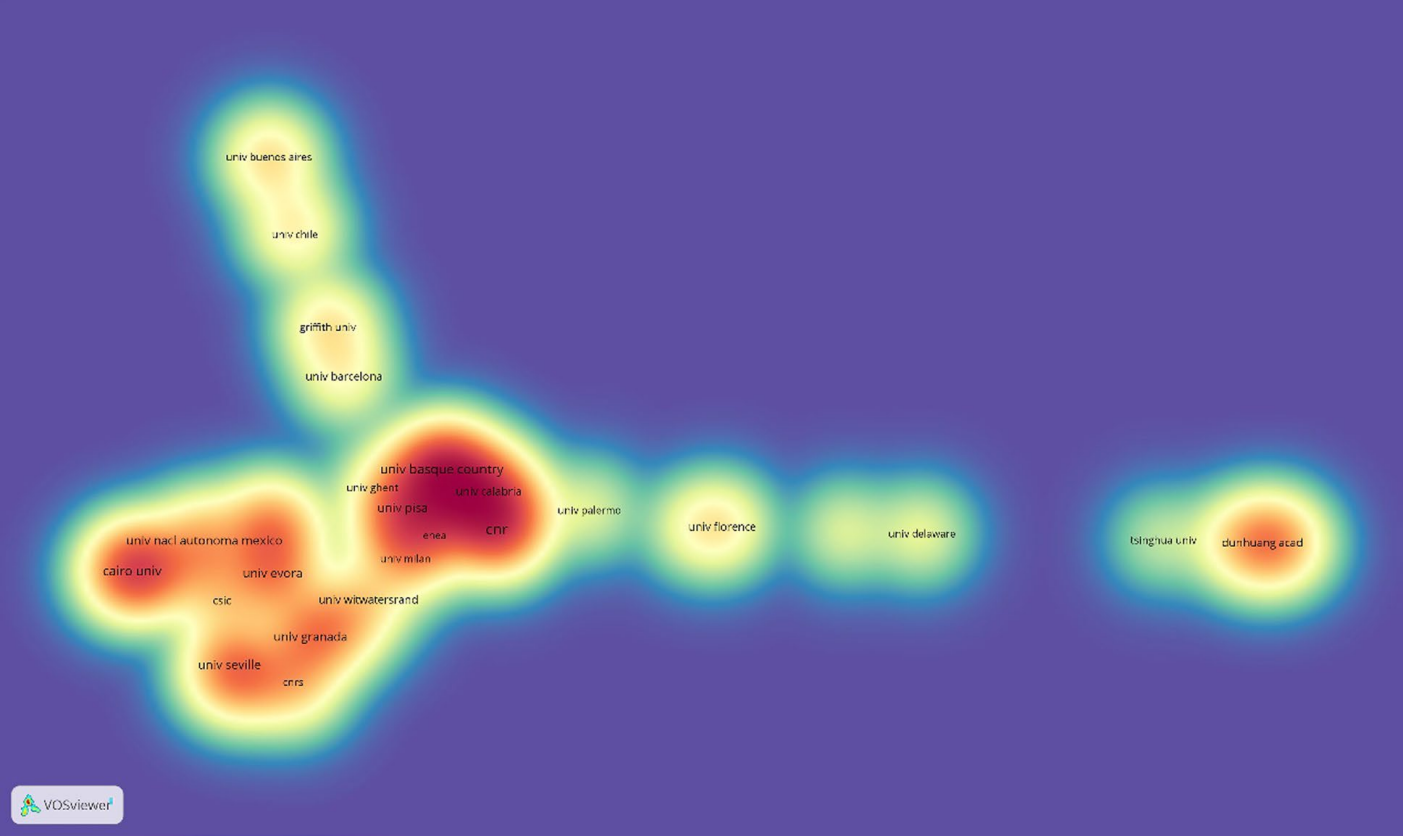
Co-institution density diagram on scientific analysis of murals (2011–2021)

In density diagram, each point has a color that indicates the density of items at that point. The redder color indicates higher number and importance of items in the neighborhood of a point. So, it can be used to observe the density of knowledge and research in a certain field. As shown in Fig. 6, the frontier of mural scientific analysis was mainly concentrated in universities and local research institutions relying on rich cultural heritage resources. Among them, CNR was the most outstanding institution, with 42 articles published. And the University of Calabria, and University of the Basque Country also stand out.

The cooperative relationships among the institutions show close links between universities. The cooperation among the University of Pisa, University of Insubria, University of Bologna, and University of Cagliari was prominent, followed by the cooperation between University of Antwerp and University of Granada, and that between Parma University and University of Calabria.

**Table 3:** Top 10 institutions on scientific analysis of murals (ranking by articles)

Ranking	Institution	Articles	Citations	Links	Total link strength
1	CNR	42	409	27	46
2	University of the Basque Country	28	148	6	15
3	Cairo University	26	331	6	18
4	University of Pisa	20	223	12	6
5	University of Evora	17	104	4	7
6	University of Granada	16	363	8	8
7	Universidad Nacional Autonoma de Mexico	15	60	7	12
8	University of Seville	15	182	6	15
9	Dunhuang Academy	14	131	6	15
10	University of Florence	14	401	5	12

Link strength is the number of publications two institutions have co-authored

#### Journal analysis

**Fig. 7:**
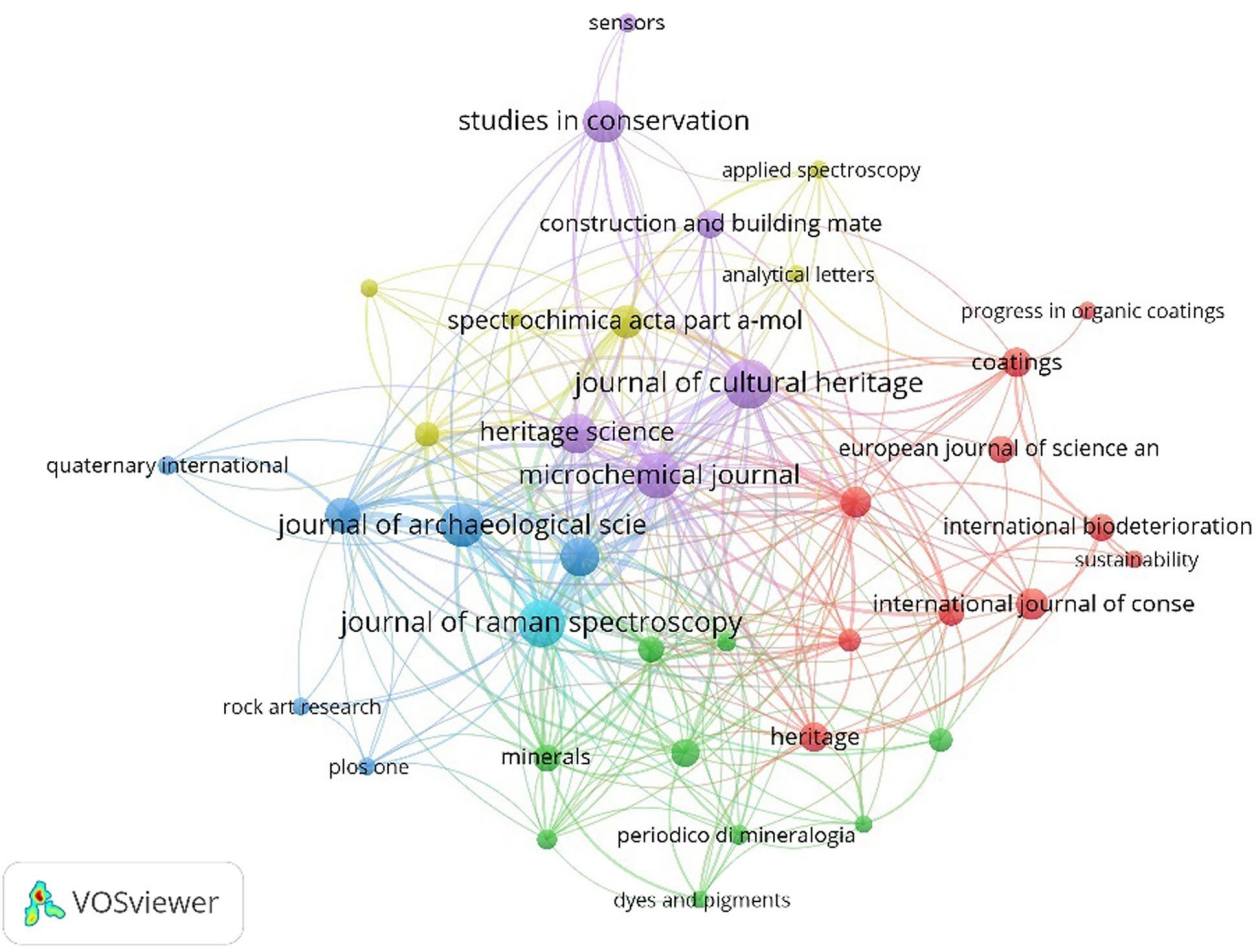
Network visualization map of citation analysis of journals publishing on scientific analysis of murals (2011–2021)

Under a threshold of published at least 5 documents per journal, 38 out of 276 journals were distinguished. Figure 7 presents the journal citation network with 38 nodes. According to Bradford’s law, if the number of papers published in a certain subject area within a journal is arranged in descending order, the journals in this subject area can be divided into three types: core area journals, related area journals, and non-relevant area journals [26]. The calculation formula is as follows:

$${\text{r}}_{0} = {\text{ }}2\ln \left( {{\text{e}}^{{\text{E}}} Y} \right)$$

where r_0_ is an estimate of how many journals should be considered to be core in a given area, E is the Euler-Mascheroni constant (0.5772) [27], and Y is the number of papers in the largest journal of the field. In this study, Y = 40. Through calculation, r_0_ can be calculated to be approximate to 8.532 and rounded to be 9. This means that at least nine journals should be considered core journals in the field of scientific analysis of murals, namely Journal of Cultural Heritage*, *Journal of Raman Spectroscopy*,* Microchemical Journal*,* Journal of Archaeological Science: Reports*,* Studies in Conservation*,* Heritage Science*,* Archaeometry*,* Archaeological and Anthropological Sciences*,* Spectrochimica Acta Part A: Molecular and Biomolecular Spectroscopy

According to Table 4, Journal of Raman Spectroscopy ranks top in citations and ranks second in the number of articles. This meant that Raman spectroscopy is by large, the most important and used technique in cultural heritage analysis. Journal of Cultural Heritage ranks top by the number of articles and ranked third in citations. Therefore, they are currently the main journals publishing the scientific analysis of mural paintings.

According to the analysis results derived from the VOSviewer software, the scientific analysis of murals spans multiple disciplines, such as chemistry, archaeology, spectroscopy, material science, engineering, and geological science. Among them, chemistry, materials science, and archaeology are the three disciplines with the largest amount of literature, indicating that the scientific analysis of wall paintings is an extensive and in-depth study concerning multiple disciplines.

**Table 4:** Nine journals in the core area of scientific analysis of murals (ranking by articles)

Ranking	Journal	Articles	Citations	Links	Total link strength
1	Journal of Cultural Heritage	40	385	29	110
2	Journal of Raman Spectroscopy	37	659	23	135
3	Microchemical Journal	35	498	31	154
4	Journal of Archaeological Science: Reports	31	200	19	83
5	Studies in Conservation	30	116	12	25
6	Heritage Science	27	167	27	84
7	Archaeometry	25	287	24	77
8	Archaeological and Anthropological Sciences	21	172	23	90
9	Spectrochimica Acta Part A: Molecular and Biomolecular Spectroscopy	18	204	19	63

Link strength is the number of co-citations by two publications of a given journal

#### Keyword co-occurrence and keyword cluster analysis

**Fig. 8:**
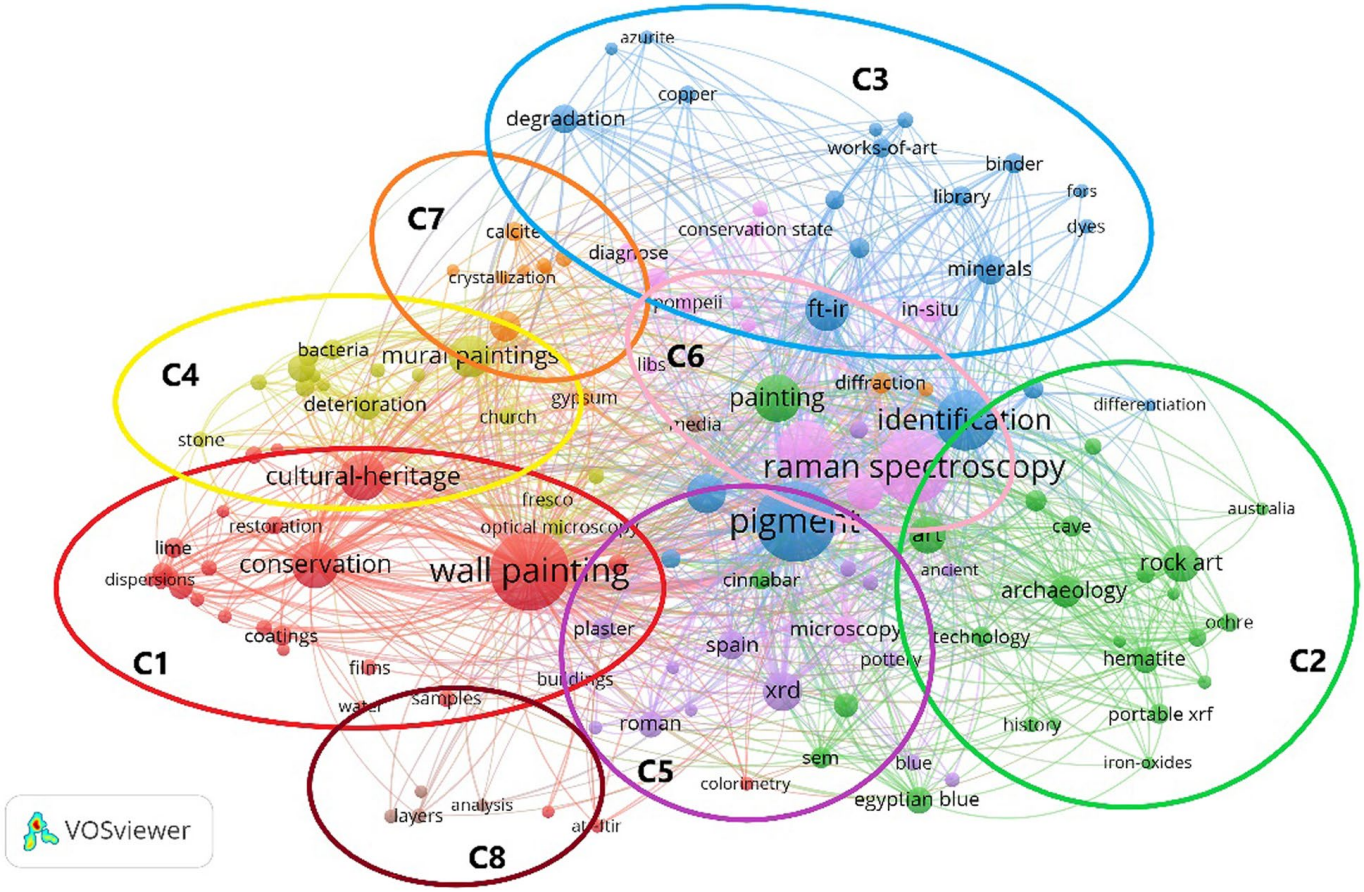
Co-occurrence network map of keywords from articles published on scientific analysis of murals (2011–2021)

**Fig. 9:**
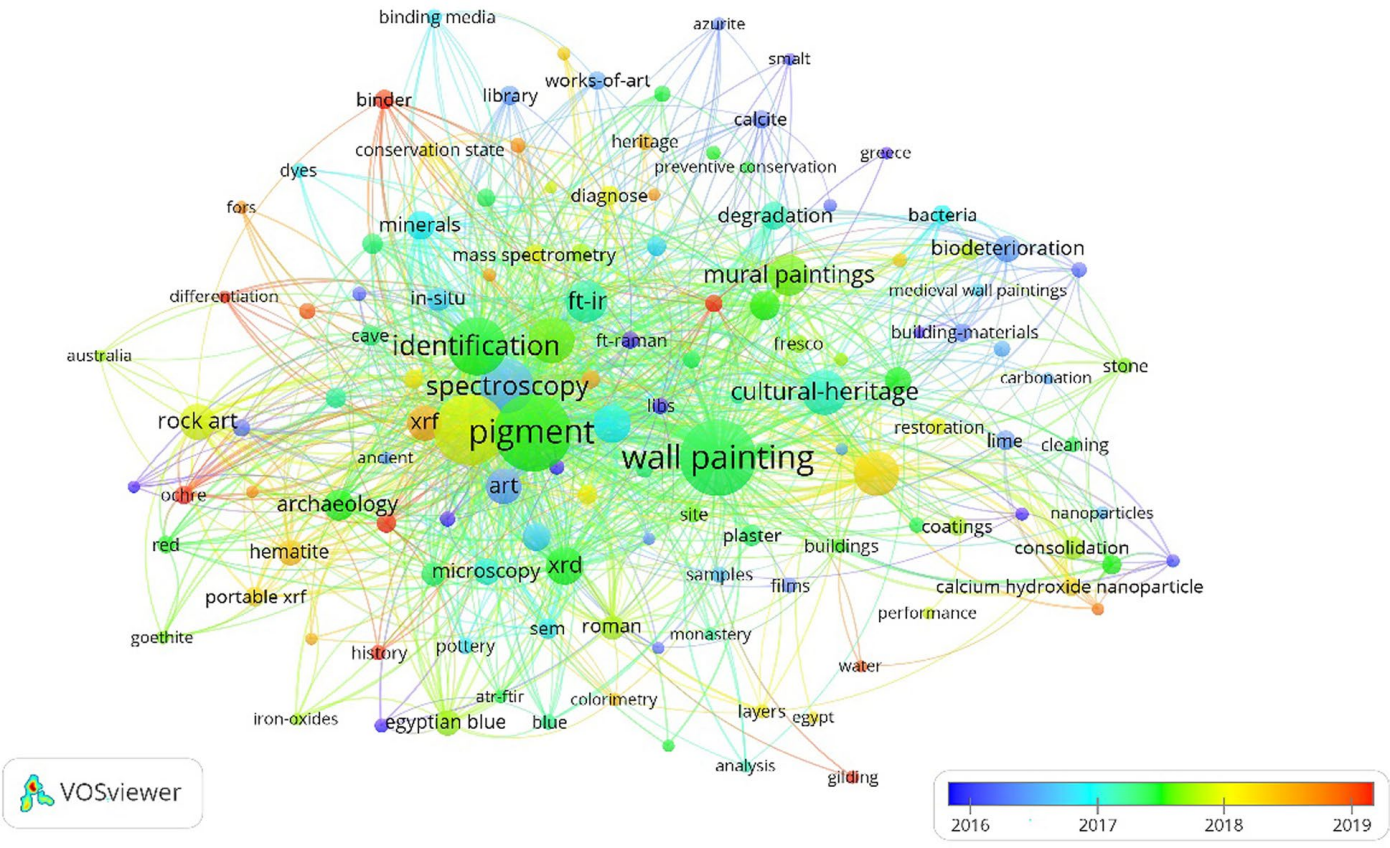
Evolution of scientific analysis of murals (2011–2021) based on the keywords

Keywords represent the core content of the literature, and high-frequency keywords effectively reflect the research hotspots in the field. As shown in Figs. 8 and 9, each keyword is represented by a node sized proportionally to its frequency. A larger number of links indicates more frequent keyword co-occurrences. The thickness of the connection reflects the strength of the connection.

Table 5 lists the top 30 high-frequency keywords. ‘pigment’ (254) appears most frequently, followed by ‘wall painting’ (244), indicating that pigment is the most important research object in this field. ‘Raman spectroscopy’, ‘spectroscopy’, ‘FT-IR’, and other keywords related to spectroscopy rank in the top 15, reflecting that spectroscopy is the most commonly used analytical method. Other high-frequency keywords, including ‘biodeterioration’, ‘degradation’, and ‘deterioration’, are related to mural biological damages and aging issues. Keywords like ‘minerals’ and ‘mortar’ are related to the component materials of wall paintings.

**Table 5:** The top 30 keywords of scientific analysis of murals (ranking by frequency)

Rank	Keyword	Frequency	Links	Total link strength
1	Pigment	254	122	1303
2	Wall painting	244	123	1128
3	Raman spectroscopy	213	120	1134
4	Identification	143	116	829
5	Spectroscopy	123	103	653
6	Painting	88	84	307
7	Cultural heritage	86	92	383
8	Conservation	85	104	414
9	FT-IR	76	88	390
10	Mural paintings	70	91	302
11	SEM-EDS	62	87	327
12	XRD	61	87	361
13	Art	55	80	279
14	Rock art	53	52	197
15	XRF	51	82	286
16	Archaeology	40	63	174
17	Mortar	37	67	168
18	Minerals	35	58	200
19	Degradation	33	50	152
20	Spain	33	63	178
21	Biodeterioration	31	45	138
22	Microscopy	31	63	183
23	Deterioration	30	54	143
24	Egyptian blue	28	48	140
25	Hematite	27	54	166
26	Roman	25	52	144
27	in-situ	24	63	149
28	Roman wall paintings	23	56	132
29	Consolidation	21	35	94
30	Plaster	20	41	81

Link strength is the number of publications in which two keywords occur together

The VOSviewer software can categorize the scattered keywords in the co-occurrence network and cluster the keywords with relatively high co-occurrence frequency. In bibliometrics, a cluster in the co-occurrence network map often represents the research theme and focus [28].

As shown in Fig. 8, the keywords are divided into 8 clusters. Each cluster or a combination of clusters represented a subfield of scientific analysis of wall paintings. The keywords in the C1 (red) cluster such as ‘conservation’, ‘calcium hydroxide nanoparticles’, ‘restoration’, ‘cleaning’, and ‘construction’, ‘preventive conservation’ in the C7 (orange) represent the research of mural conservation materials and technology. At present, scientific analysis plays an important role in the whole process of mural paintings conservation. Rosina et al. applied XRD, nuclear magnetic resonance (NMR), and IR thermography (IRT) scanning to explore the soluble salts and their transport phenomena on wall paintings [29]. Ranalli et al. assessed the cleaning effects of a new agar-gauze biogel system on mural paintings by Py-GC/MS and FT-IR [30]. Researchers comprehensively applied XRD, FTIR, scanning electron microscopy coupled with energy dispersive X-ray spectrometry (SEM-EDS) etc. to analyze the effects of calcium hydroxide nanoparticle dispersions for consolidating lime mortars [31–33]. Su et al. used FT-IR, differential scanning calorimetry (DSC), gel permeation chromatography (GPC), and SEM to evaluate the physicochemical properties of conservation materials on wall paintings [34].

The keywords ‘biodeterioration’, ‘bacteria’, ‘fungi’, ‘microorganisms’, in the C4 (yellow) cluster represent the application of scientific analysis of biodeterioration in wall paintings. Researchers applied RS, XRF, XRD, SEM to revealed the biodegradation processes on wall paintings [35]. Optical microscopy (OM) and SEM-EDS were used to study the effect of fungal hyphae growth on the cracking, flaking, and lack of cohesion of the paint layers and mortars underneath [36]. Raman spectroscopy was used to explore the effects of microbial contamination on the color alteration of mural pigments [37].

 In the C2 (green) cluster, the keywords such as ‘roman wall paintings’, ‘rock art’, ‘cave’, ‘portable XRF’, ‘prehistoric paintings’, ‘SEM’, ‘Egyptian blue’, combined with ‘Pompeii’, ‘in-situ’, ‘hyperspectral imaging’, ‘microscopy’, ‘portable Raman’ in the C6 (pink) cluster, highlighted the importance of using analysis at micro scale especially in rock art analysis but also performing in-situ analysis. And the use of portable instrumentation (mainly RS and XRF) is due to the sampling prohibition in Pompeii from the last decade. The relevant research in this area is mainly focused on the non-invasive methods and microscopic identification in t he ancient rock painting art. Researchers have adopted a multi-technique approach (OM, SEM-EDS, RS and FT-IR), aiming at a better understanding of the rock paint stratigraphy, composition, and provenance. Portable-XRF has been applied to non-invasive analyses to investigate the rock art pigments. And hand-held RS assisted with hand-held XRF were selected as the in-situ spectroscopic techniques to explore the compositions in the wall paintings [38–44] .

The ‘pigment’, ‘color’, ‘painting technique’, ‘binding media’, ‘GC-MS’ in the C3(blue) cluster, and ‘plaster’, ‘XRD’, ‘buildings’, ‘pigment identification’ in the C5 (purple) cluster, alongside with ‘media’, ‘layers’, ‘gilding’ in the C8 cluster (brown) were focused on the materials and techniques in the making of wall painting. Researchers applied SEM-EDS, Py-GC/MS, LC-ESI-MS, and cross-section analysis to explore the distribution of organic materials in different gilding layers and the gilding techniques. Techniques such as FT-IR, SEM-EDS, XRD, were adopted to analyze the material composition and explore the painting technique. RS, SEM-EDS, GC-MS were used to acquire information on the artistic materials and the painting technique prior to restoration [45–50].

The visualization shown in Fig. 9 can be expanded into the overlay visualization to illustrate the evolution of mural scientific analysis over time. Keywords from the period before 2017 include ‘pigment identification’, ‘in-situ’, ‘SEM-EDS’, ‘FT-Raman’. The wall painting research at that period was somewhat foundational and focused mainly on wall painting production materials and processes. After 2017, keywords such as ‘principal component analysis’, ‘mass-spectrometry’, ‘Ca(OH)_2_ nanoparticles’, and ‘restoration’ indicate that the development trend then was to introduce more cutting-edge analyticaland data processing methods. On the other hand, the scientific analysis was expanded to guide the research and development of mural conservation materials.

### Analysis of frequently cited literature

Ranking the articles by citations is a classic bibliometric method to reveal the most influential articles in the field [51]. Table 6 shows the top 10 frequently cited articles in mural scientific analysis from 2011 to 2021.The most frequently cited article was titled *Hydroxide nanoparticles for cultural heritage: Consolidation and protection of wall paintings and carbonate materials*, with a total number of 132 citations. This article provided an overview on the synthesis and preparation of colloidal systems tailored to the consolidation of wall paintings, and presented 2 case studies which are representative of typical consolidation problems [52]. The second most frequently cited article was entitled *A multi-technique characterization and provenance study of the pigments used in San rock art, South Africa*, with 73 citations. This study served as a starting point for the further physical analysis of southern African paints, and demonstrated the importance of using different and complimentary analytical techniques in studying the composition of wall paintings [38].

**Table 6:** The top 10 most frequently cited articles on scientific analysis of murals

Ranking	Year	Articles	Citations	Ref. No.
1	2013	Hydroxide nanoparticles for cultural heritage: Consolidation and protection of wall paintings and carbonate materials	132	[52]
2	2012	A multi-technique characterization and provenance study of the pigments used in San rock art, South Africa	73	[38]
3	2014	Identification of pigments on Byzantine wall paintings from Crete (14th century AD) using non-invasive Fiber Optics Diffuse Reflectance Spectroscopy (FORS)	71	[49]
4	2012	Studying pigments on painted plaster in Minoan, Roman and Early Byzantine Crete. A multi-analytical technique approach	70	[50]
5	2011	Spectroscopic methods for the analysis of celadonite and glauconite in Roman green wall paintings	59	[41]
6	2012	Field Raman analysis to diagnose the conservation state of excavated walls and wall paintings in the archaeological site of Pompeii (Italy)	50	[40]
7	2016	The art of building in the Roman period (89 BC-79 AD): Mortars, plasters and mosaic floors from ancient Stabiae (Naples, Italy)	48	[42]
8	2015	Invasive and non-invasive analyses for knowledge and conservation of Roman wall paintings of the Villa of the Papyri in Herculaneum	47	[43]
9	2012	In situ Raman spectroscopy analysis combined with Raman and SEM-EDS imaging to assess the conservation state of 16th century wall paintings	47	[20]
10	2014	In situ analysis with portable Raman and ED-XRF spectrometers for the diagnosis of the formation of efflorescence on walls and wall paintings of the Insula IX 3 (Pompeii, Italy)	43	[44]

## Conclusions

Bibliometric analysis results showed a growing number of researchers entering the field of wall painting scientific analysis, which generated a wealth of research views. Italy, Spain, China, the US, and France ranked the highest in the number of published articles in this field. There was relatively extensive cooperation among the top 10 countries of relevant publications. The co-cited journal network showed that the wall painting scientific analysis involved multiple disciplines, such as materials science, engineering, geology, and archaeology, which facilitated analysis in different ways, making the research more extensive and in-depth. Keyword clustering and co-occurrence networks showed that the research hotspots of mural scientific analysis mainly included evaluating the effect and mechanism of conservation materials and technologies, as well as the study of pigment composition, distribution, origin, and deterioration mechanism. In addition, future research could focus on innovations in conservation materials, and the application of novel technology and data analysis methods.

## Data Availability

All data analyzed in this study are included in the article.
